# Multi Criteria Decision Making for the Multi-Satellite Image Acquisition Scheduling Problem

**DOI:** 10.3390/s20051242

**Published:** 2020-02-25

**Authors:** Alex Elkjær Vasegaard, Mathieu Picard, Florent Hennart, Peter Nielsen, Subrata Saha

**Affiliations:** 1 Department of Materials and Production, Aalborg University, 9220 Aalborg, Denmark; aev@mp.aau.dk (A.E.V.); peter@mp.aau.dk (P.N.); 2 Airbus Defence and Space, Toulouse Area, 31555 Toulouse, France; mathieu.picard@airbus.com (M.P.); florent.hennart@airbus.com (F.H.)

**Keywords:** earth observing satellite, satellite image acquisition scheduling problem, image collection, multiple-criteria decision making, ELECTRE-III, TOPSIS, binary linear programming

## Abstract

The multi-satellite image acquisition scheduling problem is traditionally seen as a complex optimization problem containing a generic objective function that represents the priority structure of the satellite operator. However, the majority of literature neglect the collective and contemporary effect of factors associated with the operational goal in the objective function, i.e., uncertainty in cloud cover, customer priority, image quality criteria, etc. Consequently, the focus of the article is to integrate a real-time scoring approach of imaging attempts that considers these aspects. This is accomplished in a multi-satellite planning environment, through the utilization of the multi-criteria decision making (MCDM) models, Elimination and Choice Expressing Reality (ELECTRE-III) and the Technique for Order of Preference by Similarity to Ideal Solution (TOPSIS), and the formulation of a binary linear programming model. The two scoring approaches belong to different model classes of MCDM, respectively an outranking approach and a distance to ideal point approach, and they are compared with a naive approach. Numerical experiments are conducted to validate the models and illustrate the importance of criteria neglected in previous studies. The results demonstrate the customized behaviour allowed by MCDM methods, especially the ELECTRE-III approach.

## 1. Introduction

In the Satellite Image Acquisition Scheduling Problem (SIASP), the optimal schedule is defined as the set of requests that do not violate any physical constraints, while considering the numerous objectives associated with the decision-making environment [1,2,3,4]. Such objectives include maximizing customer satisfaction and maximizing the number of acquired high quality images with the consideration of the uncertainty of cloud coverage. The physical constraints include: the time windows for image acquisition of the different satellites and customer requests, maneuvering between attempts and on-board memory capacity, etc. The optimal schedule must also consider the intricate priority structure of the satellite operator, e.g., how various customer types (governmental and commercial) affect the decision-making process. The SIASP is by nature continuous, however one can use the standard scheduling approaches by considering discrete satellite paths and establishing all feasible imaging attempts for each acquisition point. As such it differs from other coverage problems [5]. Ultimately, the problem can be decomposed into two parts, scoring each attempt based on the intricate priority structure of the satellite operator and the potential quality of the image, and finding the optimal schedule based on the optimization of an objective function with the operational constraints imposed on the system.

The vast majority of the existing literature on the SIASP focuses on the second part, the optimization methodology, and in turn presents models that include manually constructed scoring procedures to enforce a certain structure on the decision process, in order to subsequently evaluate the resulting schedule on those same premises. However, manual constructions can potentially introduce a possibly damaging bias throughout the entire decision-making process as some priority structures result in image attempts with bad quality outweighing better quality attempts. This ultimately means that the satellite operator must perform an extensive correction procedure to substitute these attempts. In general these manual constructions tend to be non-transparent and perhaps even appear to be random in nature.

The solution approach in this research focuses on the first part of the decomposed problem, attempting to confront this trend and utilize models from the already established field of decision-making theory, Multi-Criteria Decision Making (MCDM) [6]. The MCDM methods explicitly evaluate conflicting alternatives based on multiple criteria in a decision-making process. Throughout this work, the two well-established MCDM methods, namely Elimination and Choice Expressing Reality (ELECTRE-III) [7] and the Technique for Order of Preference by Similarity to Ideal Solution (TOPSIS) [8] are discussed and used to score each possible acquisition attempt, while being compared with a naive scoring approach. A binary linear programming (BLP) formulation of the satellite network is then modelled to include the physical constraints, such as maneuvering, memory, payload, and stereo imaging to create the schedules. Here it should be noted that in general it is challenging to find optimal solutions to the SIASP due to the highly combinatorial nature of the scheduling and sequencing problems embedded in the SIASP. This makes real-life problem instances potentially intractable.

The remainder of this paper is organized as follows. In Section 2 the existing relevant literature is investigated, and in Section 3 the model is formulated. In Section 4 the general solution approach is introduced including the utilization of MCDM as a scoring tool. Section 5 presents the performance of the approach, and Section 6 concludes on the methodology, results, and implications. As the MCDM models ELECTRE-III and TOPSIS are already well-documented, the essential theory on the MCDM methods is presented in Appendix A.

## 2. Literature Review

Over the last two decades, image acquisition problems have been gaining enormous interest from researchers and practitioners due to the application of satellite images in various decision-making and monitoring contexts. Bensanna et al. [1] ware possibly the first researchers to formulate a mathematical model for the SIASP, designing an integer programming formulation and applying tabu search to solve the problem. Ever since, due to incredibly useful information obtained from satellite imagery in the field of meteorology, military operations, marine logistics, etc., this research stream has gained growing interest among research communities. Vasquez and Hao [2] extended the model and formulated the mathematical model as a “logic constrained” knapsack problem to obtain schedules for the satellite Spot 5. The authors used a hybrid tabu search algorithm to obtain a solution. Lemaitre et al. [9] incorporated a quality criterion and employed a combination of a greedy algorithm and dynamic programming to obtain a schedule for an agile earth observing satellite. Bianchessi et al. [10] analyzed the problem from the perspective of multiple satellites and multiple orbits. The authors also used a tabu search based heuristic and compared the quality of the solution with a column generation approach. Mansour and Dessouky [11] extended the model of Vasquez and Hao [12] and proposed a genetic algorithm based search technique for maximizing the number of images as well as profits. Jang et al. [3] developed a binary integer programming model to obtain a schedule for a Korean satellite, KOMPSAT-2. The authors used a Lagrangian relaxation approach and compared the optimal solution obtained from CPLEX solver. Tangpattanakul et al. [13] formulated the SIASP as a multi-objective optimization problem to maximize total profit and minimize the fairness of resource sharing. The authors used a random-key genetic algorithm to obtain a selection and scheduling scheme. Wang et al. [14] studied the influence of cloud coverage on the scheduling of multiple earth observation satellites. However, they neglected parameters related to weather. The authors employed a dynamic programming algorithm to obtain feasible solutions and develop heuristics for large-scale problem instances. Xu et al. [15] formulated a generalized model by considering time window constraints, resource constraints of limited on-board memory capacity, the priority level of each task, and the set up time for camera adjustment between two consecutive tasks. The authors developed an algorithm based on ant colony optimization with two heuristics for constraint handling and compared the obtained solutions with those gained from a CPLEX solver. Malladi et al. [4] proved that the SIASP is equivalent to a piece-wise linear clustered maximum weight clique problem. The authors developed a matheuristic based on a K-swap neighborhood search and tabu search algorithm to obtain a schedule. Barkaoui and Berger [16] developed a hybrid genetic algorithm to solve the multi-satellite collection scheduling problem for maximizing expected collection value. Wang et al. [17] investigated the scheduling of multiple earth observation satellites by assuming stochastic behaviour of clouds. The authors employed branch-and-price algorithm to find the schedule that maximized the profits of the satellite operator. Recently, Cui and Zhang [18] modelled the SIASP as a multi-objective optimization problem and included mission priority levels computed through TOPSIS in their objective function.

Due to the continuous technological advancements in the space industry, especially in terms of the satellites agility, and the ever-increasing demand for satellite imaging delivered with a high quality and responsiveness, the complexity of real life SIASPs will keep increasing. Our research distinguishes itself from current state of research into the SIASP, as we focus on the scoring of each attempt through an established decision-making model. This approach also serves to reduce the complexity of the optimization problem through assisting in reducing the solution space, and contributes a more robust schedule. Nine different criteria, namely area, depointing angle, sun elevation, forecasted cloud cover, customer priority, customer type, price, age of request, and weather uncertainty are considered and utilized with ELECTRE-III [19,20,21] and TOPSIS [22] to make a robust score. ELECTRE-III or TOPSIS are extensively applied in real-life decision making, and we refer to [23] for an exhaustive overview of the application aspect of these two methods.

## 3. Model Formulation

The solution approach must surmount the following challenges of the SIASP:

- Multi-criteria scoring of all feasible imaging attempts.
- Address biased optimization due to criteria weights specified by decision makers.
- Include operational constraints, e.g., maneuvering, payload specifications, memory capacity.
- Incorporate inter-dependencies in requests due to operational constraints and request specifications, such as large area or stereo imaging.
- Consider weather uncertainty.
- Find solutions in real time.

**Figure 1:**
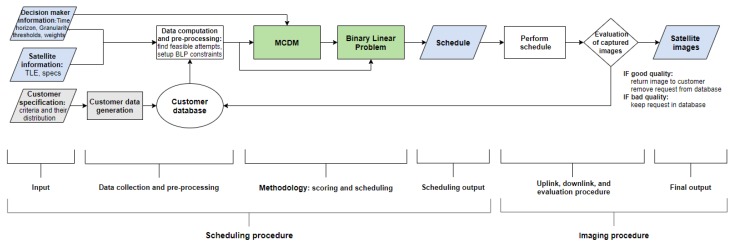
Holistic illustration of the overall solution approach for the Satellite Image Acquisition Scheduling Problem (SIASP). The data generation variables and data generation process are only required in the case of not having any data. Note that the schedule generated in the scheduling procedure is not modified by the operators prior to up-link. This is not the case in the industry.

The overall structure of the solution approach for the SIASP addressed in this research can be seen in Figure 1. This chapter discusses the elements of this structure. Note that the evaluation process illustrated in the imaging procedure of Figure 1 is neglected in this article, as an acquisition is assumed to be a completed acquisition delivered to the customer. However, we are utilizing real weather forecasts and the corresponding observed cloud cover to replicate the real-world scenario. We are only focusing on one instance and not the continuous long-term scheduling, as the goal with this paper is to show the immediate impact of a change in the scoring mechanism.

### 3.1. Input, Data Generation, and Pre-Processing

In this setup, information about decision-maker preferences, models, data generation and satellite specifications are needed to generate a scenario of satellite paths and customer requests. The information regarding the schedule horizon, and the granularity of the time segmentation (a level of discretization of the satellite path) provides an approximation of all positions where a satellite during that horizon can acquire an image. The finer the granularity, the more attempts are considered in the SIASP. Correspondingly, through the satellite specifications, customer quality thresholds, and decision maker specified granularity and time horizon, it is possible to compute all feasible attempts, and in turn gather criteria information for all attempts on sun elevation, cloud coverage forecast, depointing angle, etc. Note that for an attempt to be feasible it should satisfy the quality thresholds a customer has for each criterion and physical thresholds of the satellite’s capabilities. The most restraining feasibility threshold is the satellite’s reachability, as requests only can be acquired within a smaller time window. This reachability computation is expensive and will be discussed later. When all feasible imaging attempts are computed, a performance matrix is constructed containing all feasible attempts and their corresponding criteria. The structure for data generation of requests and schedule relative criteria is presented in Table 1. Note that the specific data consisting of integers or floats are drawn randomly from uniform distributions (U(a1;a2)), where a1 and a2 represents a minimum and maximum value, respectively.

**Figure 2:**
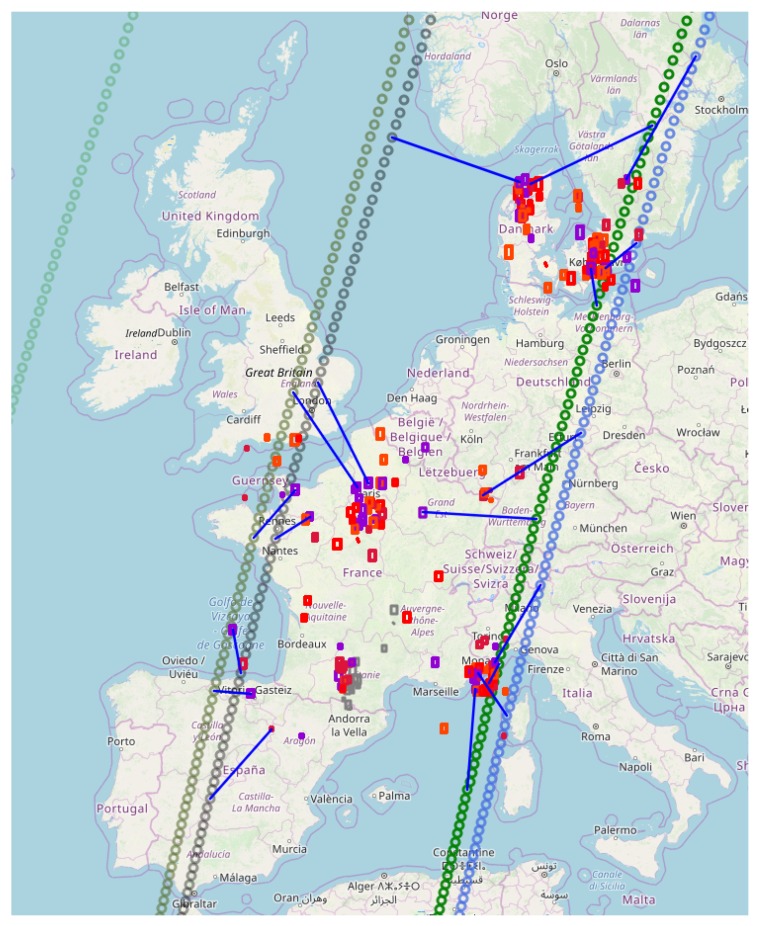
Illustration of generated scenario, i.e., 200 requests and satellite path from 2019-30-11-09-50 and 2 h ahead for SPOT 6, 7, and PLEIADES A and B, illustrated by dark green, green, blue, and black paths, respectively. The granularity is 5 seconds. Note that the request color defines its priority, and the blue lines represent an acquisition made. In addition, the performance measures for the illustrated schedule are shown in Table 4.

In this research, we considered SPOT 6 and 7, and PLEIADES A and B, as well as the capability of scheduling all these satellites by the same mission planning center. Additionally, we considered the scheduling time horizon from 2019-10-30-09-50 and 2 h ahead. During the time horizon all four satellites were able to capture images of some of the requests; see Figure 2 for an illustration of this scenario. The satellite information included satellite path information given by the Two-Line Element set (TLE) and specifications about the payload, memory, attitude maneuvering speed, etc. The TLE can be found for any publicly known satellite via the website www.n2yo.com. The TLE is a data format that constitutes a list of orbital elements for an Earth-orbiting object for a given point in time, the epoch. For analytical simplicity, we assume that the satellites have the same agility.

Customer requests are divided into two categories, namely requests from government or non-commercial organizations, and requests from commercial customers. For analytical simplicity, we assumed satellite operators only receive money from their commercial costumers, as requests from non-commercial customers could be funded in other ways, e.g., during the development of satellite system. To consider a pragmatic practice, we further classified these categories into two sub-categories, ultimately dividing the customer requests into four priority classes. In practice, commercial costumers can be classified in several ways, such as their ordering history, monetary value against each request, and their involvement in mission planning, etc. The classification, could also serve as a framework for managing emergency requests that appear due to disasters or natural calamities. After categorisation, we initiated the feasibility analysis, where non-reachable attempts and corresponding requests were removed together with requests those did not follow customer specified quality thresholds in that particular planning horizon. This offered two benefits during the scoring process, we needed to score a lower number of requests, and during schedule generation, the number of binary decision variables could be reduced significantly. Frequently, cloud coverage or sun elevation appear as significant criteria from the perspective of the customer. Therefore, these were considered during pre-processing to verify whether their respective specifications could be satisfied based on available forecast information and knowledge of satellite path. In this study, we integrated forecasted cloud coverage and its uncertainty to mimic a real problem scenario. The cloud cover data specific to the considered time horizon and locations of area of interest were obtained from www.openweathermap.org. Therefore, the real-time uncertainty was considered as a criterion in the scoring process to make sure that the schedule satisfied the cloud coverage condition. For example, if weather parameters are not considered in pre-processing and a bad quality image is captured, then the satellite resource is wasted without any fruitful outcome from the perspective of both operators and customers.

Note that we were not ignoring any requests, but this heuristic increases the system efficiency, and these infeasible requests were considered in later scheduling as presented in our computational scheme in Figure 1. Additionally, the construction of constraints for the BLP also occurs in the pre-processing. The details of the heuristics that target some of these challenges are discussed in Section 4.

### 3.2. Mathematical Optimization Problem

The SIASP considered in this research has naturally been approximated in relation to the real-world problem. The applied problem limitations are provided in Table 2.

The solution approach for the SIASP segregates the problem into two sub-problems; network and scoring, respectively. We assume the following operational challenges of the satellite scheduling to set the framework for the network sub-problem:

- The payload of each satellite can only perform one task/request at a time.
- Maneuvering time and acquisition duration make a set of attempts infeasible, i.e., some attempts cannot be performed sequentially if there is not enough time for maneuvering the attitude of the satellite. Note that if the payload tries to perform two attempts simultaneously, it will be forbidden as these two attempts are by definition part of a set of infeasible attempts.
- A request must only be acquired once within the time horizon.
- Satellites have limited memory thus affecting the schedule.
- Some requests must be acquired twice and from different angles, these are denoted as stereoscopic requests.
- Attempts must be initiated only if conditions are within some specified quality thresholds.

The focus of this research is the problem of scoring attempts, which ultimately considers contemplates the overall schedule quality. It is therefore important to note, that the score in this framework represents both the expected physical image quality connected with a certain attempt, as well as the customer satisfaction, which also represents the intricate priority structure of the satellite operator. We therefore assume that the following criteria affect the schedule quality:

- Off-nadir angle (depointing angle)
- Sun elevation angle
- The effect of real-time uncertainty on cloud coverage and observed cloud coverage
- Price
- Area
- Customer type
- Customer priority
- Age of request

These assumptions set a framework for what the mathematical optimization problem should encompass. We apply the following notation with a running index:

$$\begin{array}{cc} \; & \Gamma \;\mathrm{Set}\;\mathrm{of}\;\mathrm{satellites}\;(\mathrm{index}:\;k)\\ \; & T\;\mathrm{Set}\;\mathrm{of}\;\mathrm{satellite}\;\mathrm{positions}\;\mathrm{based}\;\mathrm{on}\;\mathrm{granularity}\;(\mathrm{index}:\;j)\\ \; & N\;\mathrm{Set}\;\mathrm{of}\;\mathrm{reachable}\;\mathrm{requests}\;\mathrm{within}\;\mathrm{scheduling}\;\mathrm{horizon}\;(\mathrm{index}:\;i) \end{array}$$

The setup of the mathematical optimization problem represents each alternative as an element in the binary variable $x_{i_{j_{k}}}$. If $x_{i_{j_{k}}}=1$, the *i*th feasible request is included in the schedule at *j*th satellite position of the *k*th satellite, and $x_{i_{j_{k}}}=0$ otherwise.

**Figure 3:**
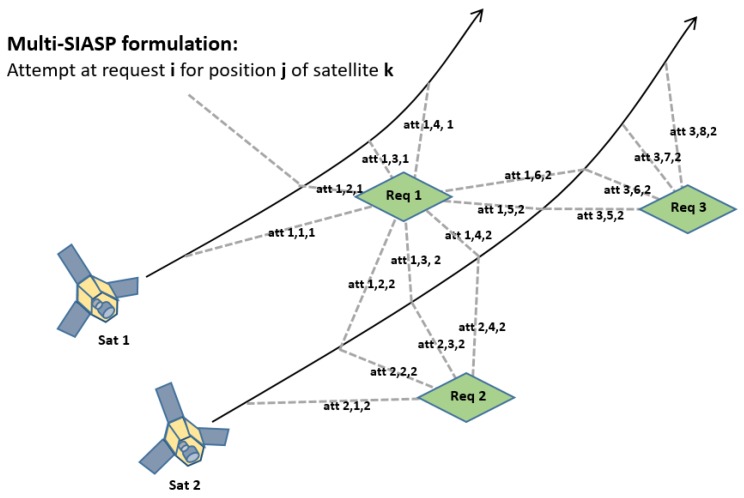
Holistic illustration of the decomposed scheduling problem and the utilized notation. Note that for a higher granularity, more satellite positions, and therefore more attempts, would be considered.

For the image acquisition schedule, although we have *N* customer requests for a particular horizon, one can eliminate some of these requests through feasibility analysis to decrease the computational complexity of the BLP problem. As it is near impossible to acquire all requests in every stated time horizon due to distance or off-nadir angle specifications, we only introduce those imaging attempts in the BLP, which comply with the feasibility analysis. The feasibility analysis does, however, make the sets N,T,Γ dependent, as only some requests are feasible from some satellite positions, motivating the subscript of Equation (1). Additionally, we order the decision variable after time (or satellite location as they are interchangeable). A representation of the SIASP with feasible attempts is shown in Figure 3.

The score for each attempt, which is computed by the MCDM, is represented by $c_{i_{j_{k}}}$. Therefore, the objective is:

(1) $$\begin{array}{cc} \; & \max_{x}\sum_{i\in N}\sum_{j\in T}\sum_{k\in \Gamma }c_{i_{j_{k}}}x_{i_{j_{k}}} \end{array}$$

The score $c_{i_{j_{k}}}$ indirectly includes the multiple-criteria related with what the decision-maker seeks to optimize. The following constraints are considered to represent physical and operational restrictions on the satellite network:

1. A request can only be acquired once.
  (2) $$\begin{array}{c} \sum_{j\in T}\sum_{k\in \Gamma }x_{i_{j_{k}}}\leq 1\;\forall i\in N \end{array}$$
2. The maneuvering between requests and the acquisition of a request must be considered while making a feasible schedule. A request acquired from a particular satellite position is considered as an attempt. Incorporating the maneuvering feasibility of the schedule involves considering the satellites consumption of time for the maneuver between that attempt and the next and the acquisition duration of the first attempt. A matrix representing all infeasible pairs of attempts is therefore pursued. Note that all attempts performed by different satellites are independent in terms of maneuverability and acquisition duration.
  To illustrate the maneuvering feasibility of attempts, we introduce additional variables representing the requests of interest (*r* and *s*, for notational convenience), and the specific satellite position where these are performed ($j_{m}$ and $j_{n}$, respectively). Thereby, investigating whether maneuvers between any two attempts performed by the same satellite is feasible. In this way, a four-dimensional matrix called an infeasibility matrix is generated for each satellite, with $F_{k}$, whose dimensions are the first request of interest (*r*); second request of interest(*s*); and the satellite position of these attempts ($j_{m}$ and $j_{n}$), where r,s∈N, $j_{m},j_{n}\in T$, and k∈Γ.
  $$F_{k}\left[r,j_{m},s,j_{n}\right]=\{\begin{array}{cc} 1 & \mathrm{if}\;T_{r_{j_{m}}\to s_{j_{n}}}^{maneuver}+T_{r}^{acquisition}>t_{j_{n}}^{clock}-t_{j_{m}}^{clock}\;\wedge \;t_{j_{n}}^{clock}\geq t_{j_{m}}^{clock}\\ 0 & \mathrm{otherwise} \end{array}$$
  where $T_{r_{j_{m}}\to s_{j_{n}}}^{maneuver}$ represents duration of maneuver between attempt $r_{j_{m}}$ to $s_{j_{n}}$, $T_{r}^{acquisition}$ is the duration of acquiring request *r*, and $t_{j_{m}}^{clock}$ is the start time of performing attempt $r_{j_{m}}$. Additionally, $T_{r_{j_{m}}}^{acquisition}$ is already computed in the pre-processing. Note, as $t_{j_{n}}^{clock}\geq t_{j_{m}}^{clock}$, we are essentially only interested in the infeasible maneuvers between attempt $r_{j_{m}}$ and future or contemporary attempts. This is because attempts are performed in a time-ordered manner, so we are only interested in which infeasible maneuvers that exist from $r_{j_{m}}$, as this ultimately represents all infeasible maneuvers present in the satellite network.
  There is a correlation between the granularity of the SIASP and satellite position, as the granularity directly affects the difference between succeeding satellite positions. However, the granularity could easily have been translated to enforce time instead of distance and thereby directly affect times of acquisition for the satellite.
  To model the $T_{r_{j_{m}}\to s_{j_{n}}}^{maneuver}$, the rotational speed of the satellite must be known. We disregard the detailed behaviour of the attitude actuators on-board the satellite, and instead assume a constant rotational speed. From industrial partners we have been informed that a good estimate for the rotational speed is 2 degrees per second. To calculate the angle of rotation between two requests, the most direct rotation is made through the plane represented by the two following vectors: vector from position of satellite at $j_{m}$ to center of request *r*; vector from position of satellite at $j_{n}$ to center of request *s*. To find these vectors we change the longitude-latitude system into the geocentric three-dimensional Cartesian coordinate system. See Appendix B.1 for further explanation. From this, we know that an infeasible maneuver for a pair of attempts $\left(r_{j_{m}},s_{j_{n}}\right)$ will result in $F_{k}\left[r,j_{m},s,j_{n}\right]=1$, while $F_{k}\left[r,j_{m},s,j_{n}\right]=0$ signifies that the maneuver between two attempts are feasible.
  Having obtained the infeasibility matrices, we now face the challenge of constructing the feasibility constraint. From Section 2 it is found that the infeasibility constraint is generally included through a pairwise infeasibility constraint or by modelling the SIASP as a maximum clique problem, see, for example [1,4]. Either way the infeasibility constraint is one of the most computationally expensive constraints to consider. As the optimization methodology is not the main focus of this research, and the exact solver is very computationally demanding in the case of either approaches of modelling the feasibility, we choose to over-constrain the solution by assuming that any subset of a set of infeasible attempts with respect to another attempt are also infeasible to each other. This will somewhat restrict the solution space from finding a solution in near real-time even for the larger problem scenarios.
  Due to the construction of $F_{k}$ matrix, the similarity of the two sets of attempts $r,j_{m}$ and $s,j_{n}$, and the fact that maneuvering between two attempts that are the same attempt must be infeasible, we know that $F_{k}\left[r,j_{m},s,j_{n}\right]=1\;\forall r,s\in N\wedge j_{m},j_{n}\in T|s=t,j_{m}=j_{n}$. Accordingly, the following must be true. For simplicity, we assume that the maneuver between a specific attempt $r_{j_{m}}$ and a single other attempt $r_{j_{m}^{\prime }}^{\prime }$ is infeasible, where $t_{j_{m}^{\prime }}^{clock}\geq t_{j_{m}}^{clock}$. That is, $F_{k}\left[r,j_{n},r^{\prime },j_{n}^{\prime }\right]=1$, and we know $F_{k}\left[r,j_{m},s,j_{n}\right]=1$ when $s_{j_{m}}$ represents the same attempt as $r_{j_{m}}$, as a consequence:
  (3) $$\begin{array}{c} \sum_{s\in N}\sum_{j_{n}\in T}F_{k}\left[r,j_{m},s,j_{n}\right]x_{s_{j_{n_{k}}}}=2, \end{array}$$
  if the two attempts $r_{j_{m}}$ and $r_{j_{m}^{\prime }}^{\prime }$ are included in the schedule. Furthermore, if additional infeasible attempts in relation to $r_{j_{m}}$ are included in the schedule, then the sum from Equation (3) will be even greater than 2. Analogously, one can observe that only performing a feasible maneuver in the framework of Equation (3) equals one, and not performing any maneuver equals zero. Based on that observation, the following infeasibility constraint is obtained, which if satisfied ensures that no maneuvers violate the physical maneuverability of the satellite.
  (4) $$\begin{array}{c} \sum_{s\in N}\sum_{j_{n}\in T}F_{k}\left[r,j_{m},s,j_{n}\right]x_{s_{j_{n_{k}}}}\leq 1\;\begin{array}{c} \forall k\in \Gamma \\ \forall r\in N\\ \forall j_{m}\in T \end{array} \end{array}$$
3. The acquisition of images take up storage on the satellites memory. The upper limit for the memory capacity devoted for the particular schedule horizon is known prior. The following formula is used for defining storage of each image.
  $$\begin{array}{c} \mathrm{Storage}\;\mathrm{size}=\frac{\mathrm{pixel}\;\mathrm{resolution}\;\times \;\mathrm{request}\;\mathrm{area}\;\times \;\mathrm{pixel}\;\mathrm{memory}\;\mathrm{consumption}}{\mathrm{compression}\;\mathrm{factor}} \end{array}$$
  Note, one pixel takes 3 bytes of memory. The upper capacity limit for the *k*th satellite is denoted, $U_{k}$, and the file size of attempt $x_{i_{j_{k}}}$ is denoted $L_{i_{j_{k}}}$.
  The image capacity constraint works as an upper limit constraint of the cumulative file size of the acquired images.
  (5) $$\begin{array}{c} \sum_{j\in T}\sum_{i\in N}L_{i_{j_{k}}}x_{i_{j_{k}}}\leq U_{k}\;\forall k\in \Gamma \end{array}$$
4. In this study, we considered the consequence where the satellite operator could deliver stereoscopic images. Given a set of requests, a subset of those requests were assumed to be stereoscopic requests. That subset was denoted λ, and for the *i*th stereoscopic request in λ, we had multiple sets of attempts that satisfied the stereoscopic imaging specifications of a convergence angle between 30 and 60 degrees. Essentially, when considering a stereo request *i*, and a first attempt at position *j* of satellite *k*, we were interested in all other second attempts $\left(j^{\prime },k^{\prime }\right)$ for the same request *i* that produced a proper convergence angle. We denote all first attempts $j_{k}$ that satisfied the conditions of a stereo request *i* by $\tau_{i}$ and all accompanying second attempts $j_{k^{\prime }}^{\prime }$ that completed a stereo request by $\tau_{i}^{\prime }\left(j,k\right)$. Note, $\tau_{i},\tau_{i}^{\prime }\left(j,k\right)\subseteq T$
  In order to represent all attempts that collectively complete this acquisition, we construct a five-dimensional binary matrix, *S*.
  $$\begin{array}{c} S\left[i,j,k,j^{\prime },k^{\prime }\right]=\{\begin{array}{cc} 1 & \mathrm{if}\;\mathrm{attempt}\;i_{j_{k}}\;\mathrm{is}\;a\;\mathrm{valid}\;\mathrm{first}\;\mathrm{attempt}\;\mathrm{of}\;\mathrm{the}\;i\mathrm{th}\;\mathrm{stereo}\;\mathrm{request}\;\mathrm{in}\;\tau_{i}\\ \; & \mathrm{in}\;\mathrm{relation}\;\mathrm{to}\;\mathrm{the}\;\sec\mathrm{ond}\;\mathrm{attempt}\;i_{j_{k^{\prime }}^{\prime }}\\ -1 & \mathrm{if}\;\mathrm{attempt}\;i_{j_{k^{\prime }}^{\prime }}\;\mathrm{is}\;a\;\mathrm{valid}\;\sec\mathrm{ond}\;\mathrm{attempt}\;\mathrm{of}\;\mathrm{the}\;i\mathrm{th}\;\mathrm{stereo}\;\mathrm{request}\;\mathrm{in}\\ & \mathrm{in}\;\tau_{i}^{\prime }\left(j,k\right)\;\mathrm{in}\;\mathrm{relation}\;\mathrm{to}\;\mathrm{the}\;\mathrm{first}\;\mathrm{attempt}\;i_{j_{k}}\\ 0 & \mathrm{otherwise} \end{array} \end{array}$$
  Therefore, the following constraint is proposed to include stereoscopic images.
  (6) $$\begin{array}{c} \sum_{k^{\prime }\in \Gamma }\sum_{j^{\prime }\in \tau_{i}^{\prime }\left(j,k\right)}S\left[i,j,k,j^{\prime },k^{\prime }\right]x_{i_{j_{k}}}=0\;\begin{array}{c} \forall k\in \Gamma \\ \forall i\in \lambda \\ \forall j\in \tau_{i} \end{array} \end{array}$$
  Note, as Equation (6) equals zero, a stereo request should not necessarily be acquired; however, if it is, the complete acquisition of the stereoscopic request must be included in the schedule for the particular planning horizon.

## 4. Solution Approach

This section details the pre-processing, as well as the utilization of MCDM as a scoring tool.

### 4.1. Reachability and Feasibility Check of Attempts

**Figure 4:**
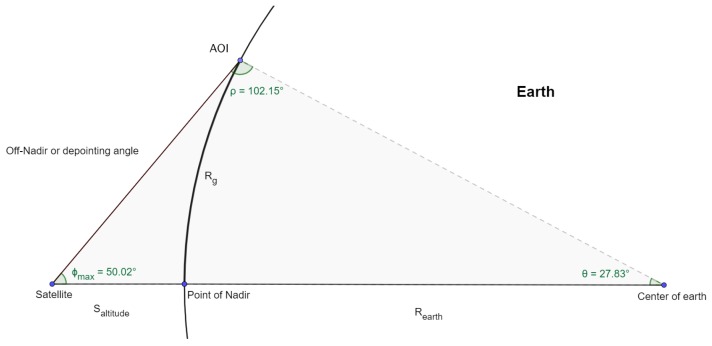
Exaggerated illustration of trigonometric relationship between maximum off nadir angle of satellite, its altitude, and the ground reachability. Practically, $\Phi_{max}=30^{{}^{\circ}}$, and the distance $S_{altitude}$ relative to $R_{earth}$ is much smaller, resulting in a much more obtuse triangle of interest.

We assume that the Earth is perfectly spherical and denote the altitude of the satellite as $S_{altitude}$. The reachability of the satellite in ground distance, $R_{g}$, can therefore be calculated from the use of sinus identity for an obtuse triangle, 180 degree property of triangles, and the arc length formula. See Equations (7)–(9) and Figure 4 for illustration.

(7) $$\begin{array}{cc} \rho & =180-sin^{-1}(\frac{sin\left(rad\left(\phi_{max}\right)\right)*\left(R_{Earth}+S_{altitude}\right)}{R_{Earth}}) \end{array}$$

(8) $$\begin{array}{cc} \theta & =180-\rho -\phi_{max} \end{array}$$

(9) $$\begin{array}{cc} R_{g} & =R_{Earth}*radians\left(\theta \right) \end{array}$$

To identify feasible attempts, we need to know the reachability of the satellite and the distances from each request to each acquisition point of the satellite. This calculation is very computationally demanding, so we apply the triangle inequality on a sphere to shortcut the process while still assuming the Earth to be a perfect sphere.

**Proposition** **1.** *In a metric space M with spherical metric d, the triangle inequality is represented as*

$$\begin{array}{c} d(a,c)\leq d(a,b)+d(b,c) \end{array}$$

*where* a,b,c *are points on the boundary of a sphere, defined in spherical coordinates as* (r,θ,ϕ) *, with a constant radius r. See [24,25].*

From the assumption of the Earth being a perfect sphere, it follows that the distance between each neighbouring acquisition position for each satellite is constant. This means that if a request is *H* times the distance between each neighbouring acquisition position away from being feasible for the satellite, the minimum number of succeeding satellite positions before a request is feasible is *H*. See Appendix B.2.

From the distance matrix, it is then possible to setup a matrix containing all feasible attempts, i.e., all distances within the $R_{g}$. We name this matrix the Performance Matrix.

### 4.2. MCDM for Scoring Acquisition Attempts

There are two reasons for MCDM being highly relevant to consider in the context of satellite scheduling. The first reason deals with the time-constrained environment of the SIASP, where MCDM is a relatively small computational task. The second reason is the contemporary comparability between attempts. When including multiple criteria for the scoring of requests, an objective function can very easily be biased, and the MCDM methods specifically aim to avoid this by evaluating attempts based on an objective that depends on that set of alternatives. Furthermore, the inclusion of additional schedule quality based criteria can be performed with ease.

In order to deduce a score from the MCDM methods, one should be aware of the conflicting perspectives of operators in the organization, as is to value different attempts relative to other attempts through a scoring procedure. It is relatively easy to select a single attempt from a set of attempts. The challenge is when one wishing to select a subset of attempts given certain constraints, because consequently it is necessary to determine the relative value of one attempt compared to another.

The TOPSIS method yields a relative closeness index, that can be used as a direct score for each attempt; that particular score does not reflect the group-wise value of different groups of attempts, but individual comparisons are still valid.

The ELECTRE-III method yields an outranking relation of all attempts relative to all other attempts stated by the credibility index, i.e., for a specific attempt, one is given *N* number of scores, where *N* is the number of other attempts, and each score represents the credibility in the hypothesis that the specific attempt outranks the other attempt. The resulting score of ELECTRE-III is the average of those scores; that is, the credibility of an attempt outranking all other attempts. Due to the use of thresholds it is easier to specify which ranges of the different criteria that has any significant meaning for the user, and thereby the ELECTRE-III approach allows the user to institute some relative value between attempts. Additionally, these thresholds allow the uncertainties inherent in a criteria evaluation to be incorporated in the decision-making environment, i.e., criterion uncertainty and sensitivity of decision maker. In this work, we utilized fixed threshold values, as opposed to an extension of the ELECTRE-III approach where the threshold value depends on the range of the criteria, e.g., in a linear manner. This could for future work especially fit the intricate priority structure of some satellite operators, where certain request types always are prioritized over others, while others are not, e.g., the treatment of emergency requests versus any other requests, and good customers versus new customers [26].

The criteria in Table 3 are included to provide the decision maker with the ability to integrate all preferences of the satellite operator, i.e., to consider both operational, priority, and quality aspects. It is clear that the quality aspect of the criteria should be included to maximize quality-related parameters, i.e., depointing angle, sun elevation, and cloud cover. Furthermore, priority, customer type, area, price and age of request were significant criteria in the intricate priority of each request to match business priorities. Ultimately utilizing all criteria gave the decision maker the problem of balancing criteria importance, but the combination of all criteria aspects yielded a much more robust score.

In the performance evaluation presented in Section 5, equal weights were utilized in all models. However, as ELECTRE-III included criteria thresholds, the weight structure was consequently very different from the other models. The threshold values presented in Table 3 are assumed to fit the preference of the satellite operator. For example, a veto threshold of 15 for cloud cover means that any attempt, which in relation to another attempt, encountered a cloud cover of 15 or worse, was never scored higher than the other attempt. In a company that encounters difficult priority structures, this is very useful for segregating certain customer requests, while at the same time considering quality.

### 4.3. GLPK Solver

To solve the proposed SIASP, we used the freely available GNU Linear Programming Kit (GLPK) package, which is widely used for large-scale mixed-integer linear programming problems. In the software package, integer restriction of decision variables is accomplished by using the branch-and-cut method [27], which integrates the cutting plane and branch-and-bound method [28,29].

## 5. Performance Analysis

### 5.1. Performance Measures Relative to Priority and Scoring Method

Another goal of this research is to analyze how customer classification, an important decision criterion for satellite operators, influences the image acquisition schedule. If we compare the results in Table 4, it can be observed in the performance of the naive or TOPSIS scoring approach that the number of acquisitions from P1 and P2 was less compared to ELECTRE-III. As mentioned earlier, satellite operators can be obligated to deliver images to non-commercial customers like government or military organizations. From that perspective, ELECTRE-III outperformed the other two approaches due to the satellite operators’ ability to set veto thresholds for attempt comparison.

Cloud coverage and depointing angle are two further aspects directly related to image quality. Table 4 likewise shows that ELECTRE-III outperformsed the other models. The total number of acquisitions was less for ELECTRE-III, but the acquired images were of good quality.

To ensure comparability, we employed equal criteria weighting across the three scoring approaches to illustrate the performance. However, each model behaved differently and a certain weighting structure in one model did not necessarily reflect a similar behavior the other models. As previously mentioned, one obvious difference in the three models was the utilization of criteria thresholds in ELECTRE-III. By employing these, the weight setting was not comparable to any of the weight settings of the other models, and in turn the performance evaluation was not either. Ultimately, it came down to which model provided the best framework for setting weights that matched the requirements of the decision maker, and in that regard ELECTRE-III provided a significantly more transparent framework for implying weights that distinguished between certain requests through their criteria information. It is very difficult to determine what weight a criteria should have in order for the model to rank in a certain way. ELECTRE-III enabled this customized ranking through its thresholds. Consequently, we are only presenting performance results for the ELECTRE-III in the next steps.

### 5.2. Running Time Relative to Granularity and Requests

For the computational performance evaluation presented in Table 5, all numerical experiments were executed on a Intel Core i5-4590 CPU with 3.30 GHz processors and 8.0 GB RAM. It can be seen in Table 5, that the computational complexity increased exponentially with the number of requests, and the effect of lowering the granularity seemed to have the same impact. The pattern was, however, not as clear, which stems from the situational effect of the model. As the number of requests increased, comparative scoring opportunities also increased, therefore there was a possibility of occurring ties among scores in the objective function. Additionally, the increasingly more complex constraints can be seen as the source of the growing time complexity.

### 5.3. Sensitivity Analysis

For the sensitivity analysis a small subset of parameters were chosen as representatives of the behaviour when modifying weights. We chose sun elevation, cloud cover and customer type, as they are important criteria in the representation of a satellite schedule. As weights must sum to 1, the range of tested weights illustrated in Table 6 means that the remaining weights sum to the residue weight. Essentially, the results illustrated what happened in the spectrum from a criteria being neglected, to it being the only considered criteria in the score. Due to the number of criteria, it is not possible to illustrate the entire grid of different weight settings. It was to be expected that an increased weight for a specific criteria would yield an increase in the performance measure linked to that criteria, and this was also what occurred, e.g., as the average observed cloud cover decreased from 12.561 to 7.11. One thing that must be remembered is that the results in Table 6 are computed on the basis of satellites acquiring requests during an effectively very short time period. The satellites each only had a window of less than 8 min to operate over the pool of customer requests, and in turn the difference in each performance measures was not as significant as could be expected, had the pool of requests been spread out further. Note that this was, however, intentional as we are interested in identifying the immediate difference in performed acquisitions.

In Table 7, we illustrate the results of modifying a single criteria threshold while fixing the rest. Again due to the number of parameters, we were only investigating one of the crucial criteria and the effects of modifying the three criteria thresholds on that. To illustrate the performance changes, 100 scenarios were created and tested. Increasing the indifference threshold did indeed make the schedule more indifferent for small improvements in cloud coverage, while it enabled all the other performance measures to be improved. In a similar way, the preference threshold determined when one specific attempt was preferred over others. It can be seen that the veto threshold had a significant impact on the ranking of the model. By utilizing a low veto threshold, the ELECTRE-III model ranked attempts with a smaller cloud cover higher, resulting in an improved observed cloud cover, but a decrease in all other performance measures.

## 6. Conclusions

This research presents a complex multi-satellite image acquisition scheduling problem and proposes a two-part system to obtain a robust image acquisition schedule. Nine different criteria are identified based on rigorous discussion with satellite operators, and MCDM techniques are used for scoring purpose for each attempt. Finally, those scores are used in the objective function of a binary linear programming formulation representing some physical constraints related with the operations. The performance and sensitivity analysis was conducted with different scenarios which demonstrate encouraging outcomes in terms of transparency of the ELECTRE-III model, as it enables the decision maker to integrate preferences in a more customized manner.

The integration of MCDM into the SIASP enables the satellite operator to focus on the many potentially conflicting sub-goals such as image quality, weather condition, satellite configuration, etc. This can significantly reduce the complexity of the problem. In this way, the final outcomes will be more passable and closer to the preferences of the satellite operator, than the weighted average of each criterion can provide. The ELECTRE-III method used in this study has the ability to consider incomparability between several attempts. The satellite operators with heterogeneous scales of measurements can choose attempts without any distortion of real information and ensure the acquisition of images from customers with higher priority. Moreover, the assimilation of ELECTRE-III is always suitable for problems where operators encounter a set of alternatives on the basis of a large set of criteria.

The main weakness of this study lies in the definition of the threshold values related to with each criterion. To some extent, the schedule performance varies with each parameter and constructing the correct parameter setting is, accordingly, a great challenge. Therefore, one of the immediate extensions of this study is the integration of stochastic multi-criteria acceptability analysis (SMAA) to explore the overall sensitivity of parameters in relation to the preferences of the satellite operator. For future research, the authors intend to propose a framework for the hierarchical structure of criteria, introducing a robust infeasibility constraint that allows all feasible attempts, as well as to integrate a more sophisticated method of handling the optimization problem. Determining the optimal threshold values and criteria weights for the ELECTRE-III method is another challenging task. In the literature, this is addressed through different approaches, e.g., by carrying out an extensive sensitivity analysis, or by the use of the methods such as regression technique or interval value fuzzy approaches [26,30]. Consequently, this also inspires a future research direction of the SIASP. Finally, it is possible to relax the capacity constraint to acquire images as much as possible, because new generations earth observation satellites may have more memory capacity and flexibility.

## Figures and Tables

**Table 1:** Structure of generated information.

Data Type	Description
Location (Area of Interest)	Uniformly distributed around Denmark and France based on longitude and latitude. The multiple subsets replicate the higher concentration of requests around e.g., urbanized areas, see Table A1.
Customer type	Assumed to be uniformly distributed on the set {1,2}. It represents the different customer types; government and commercial, respectively.
Priority	Assumed to only be dependent on customer type, i.e., customer type 1 is further divided into two segments as Priority 1 and Priority 2. Similarly, customer type 2 is divided into Priority 3 and Priority 4. In practice, it depends on the intricate priority of customers within each customer type from the space agency. For example, a four-priority class structure can be arranged as military, government, and commercial one and two.
Price	Uniform [1000, 10,000] in euros. Note that these figures are provided for the sake of simulation, but do not reflect any commercial reality. In practice, the price should be correlated with priority, commercial customer types, and location as urbanized areas have a higher demand than ocean-based requests, etc. We assume, only commercial customers to bring profit, e.g., due to collaboration, profit from government organisations are assumed to be zero.
Age	Uniform [1, 13], and it represents the days a request have been active.
Stereo request	We assume 10% of the customer requests to be stereo requests (3D), and during computation each are considered independently.
Area	Uniform [1, 1000] in km^2^, it represents the area of a particular request.
Duration of acquisition	Uniform [2, 8] in seconds. It represents the duration for a satellite to capture a specific request.

**Table 2:** Delimitation of SIASP.

Problem	Description of Limitation
Energy	The satellites energy behaviour when maneuvering and capturing images is neglected.
Satellites	Satellites are assumed to be homogeneous in terms of their camera, memory, and maneuverability specifications. Memory of 1 TB.
Agility	The maneuverability of the satellite (pitch, roll, yaw) is modeled assuming a constant angular velocity ($2^{{}^{\circ}}$ per second).
Requests	A request can be acquired with the depointing angle being directed at the center of the request. Max depointing angle of 30 degree.
Strips	We assume all requests can be acquired in a single strip. That is, we neglect decomposing requests and managing multiple strips for the acquisition of a request.
Acquisition	When acquiring a request, the attitude of the satellite is assumed to be locked in that position through the acquisition duration. It is not rotating through the acquisition.
Imaging	All satellite camera specifications are assumed to meet requests’ preferences.
Stereo	Stereoscopic imaging must be obtained from two different satellite positions with a convergence angle between $30^{{}^{\circ}}$ and $60^{{}^{\circ}}$. It can be performed by two different satellites.
Duration	For analytical tractability it is assumed that the duration is U[2,8] (in seconds). However in practice, it may be correlated with the total area of the request and satellite swath, agility, etc. For example if a satellite is equipped with pushbroom sensors, then a longer image strip requires a longer acquisition duration.
Reach	For the reachability of a request relative to a satellite, we only consider the center of that request.
Earth	Perfectly spherical.

**Table 3:** The criteria utilized in the multi-criteria decision making (MCDM) scoring. * Note, it is the observed cloud cover that one really wants to minimize, but planning is conducted based on forecasts. ** This is an ordinal criteria, where a smaller number yields a higher value.

Criteria	Objective	(Indifference/Preference/veto) Threshold Values
Area (m^2^)	max	(0/50/1000)
Depointing angle (°)	min	(2/5/40)
Sun Elevation (°)	max	(2/5/40)
Forecasted cloud coverage (%)	min *	(0/5/15)
Customer priority	min **	(0/1/2)
Customer type	min **	(0/0.5/1)
Price (monetary value)	max	(0/1000/10,000)
Age of request (days)	max	(0/1/5)
Weather uncertainty ($\sigma^{2}$)	min *	(0/2/5)

**Table 4:** The performance of different scoring approaches relative to priority. It should be noted that ELECTRE-III utilizes equal criteria weighting and the previously specified threshold values.

	TOPSIS				ELECTRE-III				Naive			
	P1	P2	P3	P4	P1	P2	P3	P4	P1	P2	P3	P4
Total #acquisitions	1	3	10	4	13	3	2	0	6	3	6	3
Total profit	0.00	0.00	77,325	22,492	0.00	0.00	18,187	0.00	0.00	0.00	42,093	14111
Avg. observed	12.000	12.000	7.600	7.250	7.230	17.333	15.000	-	6.000	12.000	12.833	5.333
cloud cover												
Total area	788.04	1901.69	6119.36	3333.36	8829.06	867.90	989.68	0.00	4572.32	1901.69	3901.24	2385.72
Avg. age	6.000	5.666	5.100	4.250	3.769	4.000	4.000	-	3.166	5.666	4.833	3.666
Avg. angle	11.598	16.473	16.271	23.570	19.637	23.558	25.917	-	14.323	16.473	18.848	16.902
Avg. sun	31.376	30.445	24.309	28.349	26.327	28.359	22.182	-	23.880	30.445	26.297	26.998
elevation												

**Table 5:** Running time for finding solution of SIASP for different scenarios in seconds. It should be noted that this includes scoring and scheduling, not the pre-processing.

Granularity\Requests	50	100	200	300	400
10	0.069	0.190	0.765	1.812	1.948
8	0.125	0.298	1.275	2.103	2.597
6	0.164	0.699	2.162	3.328	7.249
4	0.203	0.518	5.046	7.489	21.802
2	0.581	3.574	20.193	38.302	62.192

**Table 6:** This is the average performance measures for 100 generated scenarios, where the scoring through Elimination and Choice Expressing Reality (ELECTRE-III) has been conducted with different criteria weight settings. Note that the threshold values are fixed, and as the criteria weights are dependent by the sum to one constraint, this table illustrates the performance change of modifying the importance of a single criteria weight relative to all others.

Criteria	Weight	Total	P1	P2	P3	P4	Avg. Obs.	Total	Avg.	Avg.	Avg. Sun
		Acq.					Cloud Cover	Area	Age	Angle	Elevation
Sun elevation	0.0	17.58	12.85	1.05	3.24	0.44	11.235	10,168.958	4.181	21.989	26.093
	0.1	17.62	12.88	1.06	3.22	0.46	11.288	10,200.475	4.178	22.154	26.132
	0.2	17.63	12.94	1.06	3.20	0.43	11.339	10,175.572	4.191	22.329	26.168
	0.3	17.63	12.94	1.03	3.22	0.44	11.369	10,146.893	4.193	22.418	26.198
	0.4	17.67	12.92	1.08	3.24	0.43	11.368	10,114.599	4.196	22.617	26.236
	0.5	17.69	12.93	1.05	3.29	0.42	11.406	10,025.724	4.196	22.839	26.281
	0.6	17.69	12.89	1.06	3.31	0.43	11.534	9917.002	4.204	23.067	26.314
	0.7	17.71	12.83	1.05	3.41	0.42	11.707	9806.103	4.210	23.309	26.357
	0.8	17.72	12.84	1.01	3.45	0.42	11.845	9677.122	4.253	23.455	26.396
	0.9	17.72	12.80	1.01	3.50	0.41	11.984	9426.883	4.296	23.681	26.435
	1.0	18.76	5.67	4.40	5.06	3.63	22.732	9838.421	4.851	24.556	27.396
Cloud cover	0.0	17.71	12.88	1.08	3.29	0.46	12.561	10,351.571	4.149	21.825	26.167
	0.1	17.62	12.88	1.06	3.22	0.46	11.288	10,200.475	4.178	22.154	26.132
	0.2	17.61	12.84	1.06	3.26	0.45	10.699	10,125.339	4.193	22.460	26.136
	0.3	17.53	12.81	1.07	3.21	0.44	10.189	9960.712	4.206	22.624	26.127
	0.4	17.50	12.85	1.04	3.17	0.44	9.880	9813.908	4.230	22.884	26.100
	0.5	17.46	12.90	1.04	3.08	0.44	9.580	9695.274	4.238	23.056	26.095
	0.6	17.43	12.95	1.01	3.03	0.44	9.409	9543.410	4.251	23.271	26.084
	0.7	17.40	12.86	0.99	3.11	0.44	9.198	9432.141	4.287	23.529	26.072
	0.8	17.35	12.81	0.96	3.16	0.42	9.059	9270.358	4.297	23.702	26.077
	0.9	17.29	12.84	0.93	3.11	0.41	8.933	9096.544	4.326	23.902	26.077
	1.0	17.83	4.55	4.81	4.07	4.40	7.112	9000.864	5.014	23.585	26.108
Customer type	0.0	17.61	12.78	1.08	3.29	0.46	11.276	10,259.041	4.173	22.061	26.136
	0.1	17.62	12.88	1.06	3.22	0.46	11.288	10,200.475	4.178	22.154	26.132
	0.2	17.62	12.96	1.05	3.17	0.44	11.287	10,138.166	4.197	22.315	26.134
	0.3	17.63	13.00	1.05	3.14	0.44	11.331	10,031.583	4.190	22.545	26.135
	0.4	17.65	13.12	1.04	3.08	0.41	11.384	9962.152	4.213	22.714	26.132
	0.5	17.69	13.15	1.04	3.11	0.39	11.473	9901.817	4.225	22.849	26.116
	0.6	17.69	13.17	1.03	3.09	0.40	11.542	9851.040	4.234	22.939	26.111
	0.7	17.70	13.17	0.96	3.17	0.40	11.649	9786.821	4.240	23.119	26.108
	0.8	17.74	13.21	0.97	3.17	0.39	11.893	9715.841	4.237	23.214	26.110
	0.9	17.75	13.24	0.96	3.16	0.39	11.987	9637.950	4.237	23.280	26.123
	1.0	18.54	15.29	2.14	0.86	0.25	26.986	9229.606	5.038	24.385	26.100

**Table 7:** This is the average performance measures for 100 generated scenarios, where the scoring through ELECTRE-III has been conducted with different threshold settings. Note, all other parameters are fixed, i.e., equal weights and initial threshold settings, and the results illustrate performance change of modifying a single threshold value for a specific criterion. To highlight the modified threshold values, we use bold.

Criteria	(q,p,v)	Total	P1	P2	P3	P4	Avg. Obs.	Total	Avg.	Avg.	Avg. Sun
		Acq.					Cloud Cover	Area	Age	Angle	Elevation
Cloud cover	(**0.0**,5,15)	17.62	12.90	1.04	3.21	0.47	11.273	9997.886	4.167	22.161	26.132
	(**0.5**,5,15)	17.62	12.90	1.04	3.21	0.47	11.279	9997.886	4.167	22.160	26.132
	(**1.0**,5,15)	17.62	12.90	1.04	3.21	0.47	11.275	9998.242	4.166	22.164	26.132
	(**1.5**,5,15)	17.62	12.90	1.04	3.21	0.47	11.282	9999.406	4.167	22.164	26.135
	(**2.0**,5,15)	17.62	12.91	1.04	3.20	0.47	11.300	9999.536	4.164	22.166	26.130
	(**2.5**,5,15)	17.63	12.92	1.04	3.20	0.47	11.329	10,002.442	4.166	22.170	26.133
	(**3.0**,5,15)	17.63	12.92	1.04	3.20	0.47	11.332	10,008.931	4.169	22.166	26.132
	(**3.5**,5,15)	17.63	12.92	1.04	3.20	0.47	11.340	10,004.778	4.168	22.170	26.131
	(**4.0**,5,15)	17.63	12.93	1.03	3.20	0.47	11.351	10,006.516	4.173	22.162	26.131
	(**4.5**,5,15)	17.63	12.93	1.03	3.20	0.47	11.366	10,006.516	4.173	22.159	26.131
Cloud cover	(0,**0**,15)	17.62	12.86	1.04	3.26	0.46	11.054	9991.244	4.168	22.197	26.135
	(0,**1.5**,15)	17.62	12.86	1.04	3.25	0.47	11.078	10,004.304	4.168	22.189	26.134
	(0,**3.0**,15)	17.62	12.89	1.04	3.22	0.47	11.202	10,008.823	4.168	22.142	26.128
	(0,**4.5**,15)	17.62	12.90	1.04	3.21	0.47	11.265	10,001.395	4.168	22.159	26.133
	(0,**6.0**,15)	17.63	12.92	1.04	3.20	0.47	11.346	9998.861	4.165	22.161	26.132
	(0,**7.5**,15)	17.65	12.92	1.04	3.22	0.47	11.430	10,014.056	4.163	22.145	26.135
	(0,**9.0**,15)	17.64	12.95	1.04	3.20	0.45	11.503	10,019.136	4.180	22.141	26.134
	(0,**10.5**,15)	17.64	12.94	1.04	3.21	0.45	11.559	10,037.506	4.176	22.156	26.132
	(0,**12.0**,15)	17.65	12.98	1.04	3.18	0.45	11.700	10,046.744	4.172	22.146	26.134
	(0,**13.5**,15)	17.65	13.02	1.04	3.15	0.44	11.791	10,063.827	4.174	22.154	26.135
Cloud cover	(0,5,**7.50**)	17.38	12.73	1.04	3.19	0.42	9.597	9743.103	4.212	22.313	26.114
	(0,5,**9.75**)	17.49	12.76	1.05	3.24	0.44	10.109	9805.737	4.197	22.324	26.129
	(0,5,**12.00**)	17.55	12.80	1.05	3.25	0.45	10.564	9868.942	4.190	22.218	26.141
	(0,5,**14.25**)	17.62	12.85	1.05	3.26	0.46	11.132	9980.082	4.169	22.196	26.130
	(0,5,**16.50**)	17.65	12.95	1.05	3.17	0.48	11.577	10,053.108	4.170	22.139	26.138
	(0,5,**18.75**)	17.72	13.04	1.06	3.19	0.43	12.114	10,063.496	4.167	22.095	26.164
	(0,5,**21.00**)	17.75	13.04	1.06	3.22	0.43	12.494	10,125.880	4.168	21.937	26.166
	(0,5,**23.25**)	17.78	13.15	1.01	3.20	0.42	13.007	10,099.129	4.149	21.896	26.169
	(0,5,**25.50**)	17.82	13.24	1.00	3.21	0.37	13.535	10,119.845	4.138	21.923	26.172
	(0,5,**27.75**)	17.85	13.23	1.02	3.26	0.34	13.842	10,118.423	4.143	21.910	26.191

**Figure A1:**
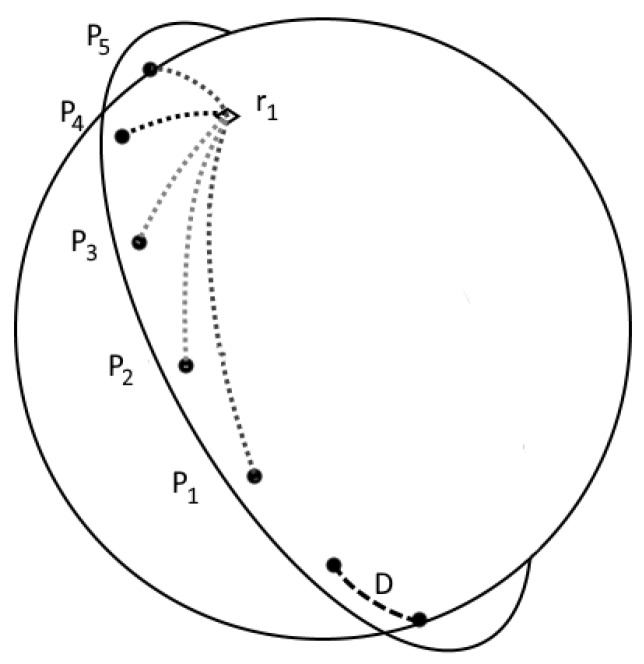
Illustration of distance calculation for acquisition points to request.

## Appendix A. Multi-Criteria Decision Making

It is very common to be confronted with decision-making problems which involve multiple perspectives on assessing the actions they concern. These perspectives usually form the criteria of evaluation by mapping them with a value function. Generally, we refer to these kinds of problems as multi-criteria decision making (MCDM) problems. To aid the decision makers in solving this class of problems, a suite of methods have been proposed in the literature, e.g., AHP, TOPSIS, PROMETHEE, ELECTRE, VIKOR, etc. A more exhaustive set of methods with their selection criteria in the context of decision making scenarios has been illustrated in the recent study.

Based on the SIASP problem characteristics and available information, in this study, we consider two prominent and widely used MCDM methods, specifically ELECTRE-III and TOPSIS. To illustrate these methods, we will consider a finite set of alternatives $A=\{a_{1},a_{2},\ldots ,a_{m}\}$ that are required to be ranked based on a family of coherent criteria $\left\{g_{1},g_{2},\ldots ,g_{n}\right\}$. The performance of the alternative $a_{i}$ with respect to the criterion $g_{j}$ is given by the value function $g_{j}\left(a_{i}\right)$.

### Appendix A.1. ELECTRE-III

ELECTRE [4,6] is an abrreviation for Elimination and Choice Expressing Reality, and there are multiple versions focusing on either selection, ranking, or sorting. ELECTRE belongs to the outranking class of MCDM, and mainly consists of two phases; first, the construction of one or several outranking relations, which in a comprehensive way compares each pair of information; second, an exploitation procedure that analyses the recommendations obtained in the first phase.

ELECTRE-III [3] is designed to incorporate the fuzzy nature of decision making by including thresholds of indifference and preference, which makes it possible for decision makers to influence not only the importance of criteria but also to incorporate range of information to the analysis. This setup enables the introduction of an incomparability feature, which occurs when there is no evidence in favor of either alternatives, a feature other models neglect. Another interesting feature is that ELECTRE-III is fundamentally non-compensatory, which makes bad scores more important than for other types of MCDM methods. To understand the first phase of ELECTRE-III, two concepts must be introduced; thresholds and outranking.

In the ELECTRE III framework of preference modelling, we need to introduce the criteria threshold of preference ($p_{j}$), indifference ($q_{j}$), and veto ($v_{j}$). The indifference threshold is the maximum difference between *a* and *b* on $g_{j}$ compatible with them being indifferent for the $g_{j}$ criteria; the preference threshold is the minimum difference between $a_{i_{1}}$ and $a_{i_{2}}$ on $g_{j}$ compatible with the preference of one over the other on $g_{j}$; the veto threshold is the minimum difference for $a_{i_{1}}$ over $a_{i_{2}}$ on $g_{j}$ incompatible with *b* being preferred to *a* on any other $g_{j}$, i.e., disregarding all other evidence of alternative $a_{i_{2}}$ being at least as preferred as alternative $a_{i_{1}}$. Note, $v_{j}\geq p_{j}\geq q_{j}$, and for simplicity we assume that thresholds are not dependent on the performance of alternatives.

ELECTRE-III [1] builds a fuzzy outranking relation on the set of alternatives, representing credibility for the hypothesis that an alternative is at least as good as another one. The construction of this relation involves concordance and discordance indices, where the concordance index represents the strength of the coalition of criteria being in favor of the hypothesis, while the discordance index represents the opposite set of criteria being discordant with the hypothesis. To perform tests of these hypothesis, i.e., to estimate the credibility of outranking, it is necessary to aggregate the concordance and discordance for each pair of alternative $\left(a_{i_{1}},a_{i_{2}}\right)\in A^{2}$ in the following way:

- The concordance index for each criterion $g_{j}$,

$$\begin{array}{c} \varphi_{t}\left(a_{i_{1}},a_{i_{2}}\right)=\{\begin{array}{cc} 1 & \mathrm{if}\;g_{j}\left(a_{i_{2}}\right)-g_{j}\left(a_{i_{1}}\right)\leq q_{j}\\ \frac{p_{j}-\left[g_{j}\left(a_{i_{2}}\right)-g_{j}\left(a_{i_{1}}\right)\right]}{p_{j}-q_{j}} & \mathrm{if}\;q_{j}<g_{j}\left(a_{i_{2}}\right)-g_{j}\left(a_{i_{1}}\right)<p_{j}\\ 0 & \mathrm{if}\;p_{j}\leq g_{j}\left(a_{i_{2}}\right)-g_{j}\left(a_{i_{1}}\right) \end{array} \end{array}$$

- The discordance index for each criterion $g_{j}$,

$$\begin{array}{c} d_{j}\left(a_{i_{1}},a_{i_{2}}\right)=\{\begin{array}{cc} 1 & \mathrm{if}\;g_{j}\left(a_{i_{2}}\right)-g_{j}\left(a_{i_{1}}\right)\geq v_{j}\\ \frac{\left[g_{j}\left(a_{i_{2}}\right)-g_{j}\left(a_{i_{1}}\right)\right]-p_{j}}{v_{j}-p_{j}} & \mathrm{if}\;v_{j}>g_{j}\left(a_{i_{2}}\right)-g_{j}\left(a_{i_{1}}\right)>p_{j}\\ 0 & \mathrm{if}\;p_{j}\geq g_{j}\left(a_{i_{2}}\right)-g_{j}\left(a_{i_{1}}\right) \end{array} \end{array}$$

- The credibility index representing the degree of outranking for each criterion $g_{j}$. Note, that $\Phi \left(a_{i_{1}},a_{i_{2}}\right)=\frac{1}{k}\sum_{j=1}^{r}k_{j}\varphi_{j}\left(a_{i_{1}},a_{i_{2}}\right)$, where $k=\sum_{j=1}^{r}k_{j}$.
  σ(ai1,ai2)={Φ(ai1,ai2)0000ifdj(ai1,ai2)≤Φ(ai1,ai2)∀j∈{1,2,…,r}∏j∈JΦ(ai1,ai2)(1−dj(ai1,ai2))1−Φ(ai1,ai2)otherwise
  where $J=\{j\in \left\{1,2,\ldots ,r\right\}:d_{j}\left(a_{i_{1}},a_{i_{2}}\right)>\Phi \left(a,a_{i_{2}}\right)\}$. If the discordance index for *a* relative to *b* is 1 for any criteria, the credibility index is then 0, representing no confidence in the hypothesis that $a_{i_{1}}$ is as least as good as $a_{i_{2}}$.

The second phase of ELECTRE-III is the ranking of alternatives relative to the credibility index obtained in the first phase. The ranking is obtained as the intersection of two complete preorders, that are the results of two algorithms sorting them in either descending or ascending order. These will not be described in this document, due to the way the solution approach utilizes the ELECTRE method in Section 4.

### Appendix A.2. TOPSIS

Technique of Order Preference by Similarity to Ideal Solution (TOPSIS) [7] is one of the prominent methods based on the concept of ideal points. It aids decision makers in selecting the alternative which should have the shortest distance from positive ideal solution (PIS) and the farthest distance from negative ideal solution (NIS).

The TOPSIS method requires two kinds of information from the user to find the ranking of the alternative. One is decision information (often we call it as decision matrix) of the alternatives against a predefined set of criteria and that can be summarized in the following matrix:

$$D=\begin{array}{ll} & \begin{array}{cccc} \;\;g_{1} & \;\;\;g_{2} & \;\cdots & \;g_{n} \end{array}\\ \begin{array}{l} a_{1}\\ a_{2}\\ \vdots \\ a_{m} \end{array} & \left(\begin{array}{llll} g_{1}\left(a_{1}\right) & g_{2}\left(a_{1}\right) & \cdots & g_{n}\left(a_{1}\right)\\ g_{1}\left(a_{2}\right) & g_{2}\left(a_{2}\right) & \cdots & g_{n}\left(a_{2}\right)\\ \vdots & \vdots & \vdots & \cdots \\ g_{1}\left(a_{m}\right) & g_{2}\left(a_{m}\right) & \cdots & g_{n}\left(a_{m}\right) \end{array}\right) \end{array}$$

Other information is the relative importance/weights of the criteria. Let *w* = (*w*_1_,*w*_2_,…*w*_n_) be the weights of the criteria. Based on the above information, TOPSIS can rank the alternatives by using the following steps:

- Calculate normalized decision matrix $R={\left(r_{ij}\right)}^{m\times n}$, representing the normalized criteria values.
  $$r_{ij}=\frac{g_{j}{\left(a_{i}\right)}^{\pm }}{\sqrt{\sum_{i=1}^{m}{\left(g_{j}\left(a_{i}\right)\right)}^{\pm 2}}}$$
  where − sign and + sign are used for the cost criteria and benefit criteria, respectively.
- Calculate the weighted normalized decision matrix $V={\left(v_{ij}\right)}^{m\times r}$
  $$v_{ij}=r_{ij}\times w_{j}\;\mathrm{for}\;i\in \left\{1,2,\ldots ,m\right\}\wedge j\in \left\{1,2,\ldots ,n\right\}$$
- Determine the positive ideal and negative ideal solutions $A^{*}$ and $A^{-}$:
  $$\begin{array}{cc} A^{*} & =\left\{v_{1}^{*},v_{2}^{*},\ldots ,v_{r}^{*}\right\},\;\mathrm{where}\;v_{j}^{*}=max_{i}\left(v_{i,j}\right)\\ A^{-} & =\left\{v_{1}^{-},v_{2}^{-},\ldots ,v_{r}^{-}\right\},\;\mathrm{where}\;v_{j}^{-}=min_{i}\left(v_{ij}\right) \end{array}$$
- Calculate the Euclidean distances of each alternative to the ideal solutions.
  $$\begin{array}{c} d_{i}^{*}=\sqrt{\sum_{j=1}^{r}{\left(v_{ij}-v_{j}^{*}\right)}^{2}}\;\mathrm{and}\;d_{i}^{-}=\sqrt{\sum_{j=1}^{r}{\left(v_{ij}-v_{j}^{-}\right)}^{2}} \end{array}$$
  for i={1,2,…,m}
- Calculate the relative closeness coefficient of each alternative to the ideal solutions, *CC_i_*.
  $$\begin{array}{c} CC_{i}=\frac{d_{i}^{-}}{d_{i}^{+}+d_{i}^{-}} \end{array}$$
- Rank the alternatives according to the relative closeness to the ideal solution. The *CC_i_* measures the ratio of the total Euclidean distance to ideal solutions coming from the distance to the negative ideal. The bigger the *CC_i_*, the higher the rank.

## Appendix B. Additions to Solution Approach

### Appendix B.1. Transformation of Vector System

Note that the longitude-latitude system basically is a spherical coordinate system, where the radius *R* in the system is the Earth’s radius. The conversion is therefore simple and represented by

$$\begin{array}{cc} x & =R*Cos(latitude)*Sin(longitude)\\ y & =R*Cos(latitude)*Sin(longitude)\\ z & =R*Sin(latitude) \end{array}$$

Note, for the satellites Cartesian coordinate the satellite altitude is added to the radius, *R*. The vectors of interest are represented by the depointing angle of both attempts, and the effective angle, between these two vectors is found by $cos^{-1}\left(\frac{v_{1}*v_{2}}{|v_{1}\left|*\right|v_{2}|}\right)$. As mentioned in the beginning of the section, the rotational speed is given, and duration of rotation can be calculated as $\frac{\mathrm{effective}\;\mathrm{angular}\;\mathrm{measure}}{\mathrm{rotational}\;\mathrm{speed}}$.

### Appendix B.2. Reachability of Requests

**Example** **1.**
*In the illustration in Figure A1, the distance from* $P_{1}$ *to* $r_{1}$ *is denoted* $d(P_{1},r_{1})$ *. In addition,* d(Pn,Pn+1)=D0∀n *. Note that the distance is calculated in ground distance from the nadir points. Additionally, ground distance is calculated s.t.* $D\left(P_{n},r_{m}\right)\leq \pi R_{earth}\;\forall P_{n}\in T\;and\;\forall r_{m}\in N$ *, where* $R_{earth}$ *is the radius of earth. The satellites ground level reachability is denoted* $R_{g}$ *. We assume* $d\left(P_{1},r_{1}\right)-R_{g}\geq H*D$ *, i.e., the distance until reachability can be expressed as at least H times the distance between acquisition points.*

*The inequality given bellow is always satisfied:*

$$\begin{array}{c} d\left(P_{1},r_{1}\right)\leq d\left(P_{1},P_{2}\right)+d\left(P_{2},r_{1}\right) \end{array}$$

*Due to the assumption of the Earth being a perfect sphere, we can represent* d(Pn,Pn+1)=D0∀n *, and a generalization of the relationship can be expressed as:*

$$\begin{array}{c} d\left(P_{1},r_{1}\right)\leq nD+d\left(P_{1+n},r_{1}\right) \end{array}$$

*Note,* $d(P_{1+n},r_{1})$ *is unknown for* n≥1 *. However, qua the assumption and the generalization, we know*

$$\begin{array}{cc} HD & \leq d\left(P_{1},r_{1}\right)-R_{g}\\ & ↓\\ HD\leq d\left(P_{1},r_{1}\right)-R_{g} & \leq nD+d\left(P_{1+n},r_{1}\right)-R_{g}\\ & ↓\\ HD & \leq nD+d\left(P_{1+n},r_{1}\right)-R_{g} \end{array}$$

*For* n=H *:*

$$\begin{array}{cc} HD & \leq HD+d\left(P_{1+H},r_{1}\right)-R_{g}\\ & ↓\\ R_{g} & \leq d(P_{1+H},r_{1}) \end{array}$$

*Yielding that neighbouring acquisition point number H after the acquisition point* $P_{1}$ *is may be potentially able to reach the request. All neighbours* n≤H *is definitely not able to reach the request, and the calculation of* $d(P_{1+n},r_{1})$ *can therefore be omitted for all* n<H *.*

### Appendix B.3. Data Input

**Table A1:** Scheme for generating the location of requests. Uniform distributions to and from for both lattitude and longitude. Information gathered from www.latitudelongitude.org.

Location	Latitude	Longitude
Copenhagen	55;56	12;13
Aalborg	56.5;57.5	9;10
Entire Denmark	54.769;57.72	8.24;14.70
Toulouse	43;44	1;2
Paris	48;49.5	1.5;3
Nice	43;44	7;8
Entire France	41.59;51.0	−4.65;9.45

**Table A2:** Presentation of all inserted information in the system.

Variable	Description
Start schedule	Date and time of scheduling start.
Hours ahead	Defining scheduling horizon.
Granularity	Defining the time segmentation of the satellite path.
TLEs	Two-line element set defining the satellite path. Note that these should be updated accordingly.
Maximum off-nadir angle	Determining the maximum depointing angle a satellite image can be acquired with without its quality being too bad. Set to $30^{{}^{\circ}}$.
Satellite altitude	Set to 645, but could be extracted from TLEs.
Earth Radius	6,371,230 m.
Rotation speed	Camera rotation speed. Set to $2^{{}^{\circ}}$/s.
Camera resolution	Set to 1 m^2^/pixel.
Capacity limit of satellite	Set to 1 TB.
Data location	See Table A1.
Schedule relative criteria	See Table 1.
